# Immune responses to SARS-CoV-2 in three children of parents with symptomatic COVID-19

**DOI:** 10.1038/s41467-020-19545-8

**Published:** 2020-11-11

**Authors:** Shidan Tosif, Melanie R. Neeland, Philip Sutton, Paul V. Licciardi, Sohinee Sarkar, Kevin J. Selva, Lien Anh Ha Do, Celeste Donato, Zheng Quan Toh, Rachel Higgins, Carolien Van de Sandt, Melissa M. Lemke, Christina Y. Lee, Suzanne K. Shoffner, Katie L. Flanagan, Kelly B. Arnold, Francesca L. Mordant, Kim Mulholland, Julie Bines, Kate Dohle, Daniel G. Pellicci, Nigel Curtis, Sarah McNab, Andrew Steer, Richard Saffery, Kanta Subbarao, Amy W. Chung, Katherine Kedzierska, David P. Burgner, Nigel W. Crawford

**Affiliations:** 1 Department of Paediatrics, The University of Melbourne, Melbourne, Victoria Australia; 2 Infection and Immunity, Murdoch Children’s Research Institute, Melbourne, Australia; 3 Department of General Medicine, The Royal Children’s Hospital, Melbourne, Victoria Australia; 4 Department of Microbiology and Immunology, Peter Doherty Institute for Infection and Immunity, The University of Melbourne, Melbourne, Victoria Australia; 5 Department of Hematopoiesis, Sanquin Research and Landsteiner Laboratory, Amsterdam UMC, University of Amsterdam, Amsterdam, Netherlands; 6 Department of Biomedical Engineering, University of Michigan, MI, USA; 7 Department of Infectious Diseases, Launceston General Hospital, Launceston, Tasmania Australia; 8 School of Health Sciences and School of Medicine, University of Tasmania, Launceston, Tasmania Australia; 9 Department of Immunology and Pathology, Monash University, Commercial Road, Melbourne, Victoria Australia; 10 School of Health and Biomedical Science, RMIT University, Melbourne, Victoria Australia; 11 Infectious Diseases Unit, Department of General Medicine, The Royal Children’s Hospital, Melbourne, Australia; 12 Department of Gastroenterology, The Royal Children’s Hospital, Melbourne, Victoria Australia; 13 WHO Collaborating Centre for Reference and Research on Influenza, Melbourne, Australia

**Keywords:** Immunology, SARS-CoV-2, Paediatric research

## Abstract

Compared to adults, children with severe acute respiratory syndrome coronavirus 2 (SARS-CoV-2) have predominantly mild or asymptomatic infections, but the underlying immunological differences remain unclear. Here, we describe clinical features, virology, longitudinal cellular, and cytokine immune profile, SARS-CoV-2-specific serology and salivary antibody responses in a family of two parents with PCR-confirmed symptomatic SARS-CoV-2 infection and their three children, who tested repeatedly SARS-CoV-2 PCR negative. Cellular immune profiles and cytokine responses of all children are similar to their parents at all timepoints. All family members have salivary anti-SARS-CoV-2 antibodies detected, predominantly IgA, that coincide with symptom resolution in 3 of 4 symptomatic members. Plasma from both parents and one child have IgG antibody against the S1 protein and virus-neutralizing activity detected. Using a systems serology approach, we demonstrate higher levels of SARS-CoV-2-specific antibody features of these family members compared to healthy controls. These data indicate that children can mount an immune response to SARS-CoV-2 without virological confirmation of infection, raising the possibility that immunity in children can prevent the establishment of SARS-CoV-2 infection. Relying on routine virological and serological testing may not identify exposed children, with implications for epidemiological and clinical studies across the life-span.

## Abstract (web summary)

Children with SARS-CoV-2 infection are more likely to have mild symptoms and may be asymptomatic, but underlying reasons remain unclear. Here, the authors show cellular, cytokine and antibody response to SARS-CoV-2 infection in three children who repeatedly tested negative for the virus by PCR, despite high exposure in the household.

## Introduction

To date, children represent a small proportion of SARS-CoV-2 confirmed coronavirus disease (COVID-19) cases^1–3^. Children are predominantly infected from symptomatic household adult contacts^4,5^. Children have comparatively milder COVID-19 disease and up to one-third are asymptomatic^6^. The immunological basis for milder pediatric disease is unclear, but may be relevant to other viral pandemics where striking age-related epidemiological differences were observed^7^. In SARS-CoV-2 infection, reduced respiratory epithelial expression of the ACE2 receptor and trained innate immunity in children have been proposed^8,9^. Investigating immune responses to SARS-CoV-2 across all age groups is key to understanding disease susceptibility, severity determinants, and vaccine candidates. Detailed investigations of immune responses during SARS-CoV-2 infection have been reported in adults^10–12^, with exposure to SARS-CoV-2 causing specific T cell responses without seroconversion^13^. Data on immune responses in children exposed to SARS-CoV-2 are limited.

Here, we show that three children repeatedly exposed to SARS-CoV-2 in their household mount cellular and antibody-mediated immune responses similar to their infected parents and specific to SARS-CoV-2, without virological confirmation of infection.

## Results

### Patient Characteristics

**Fig. 1:**
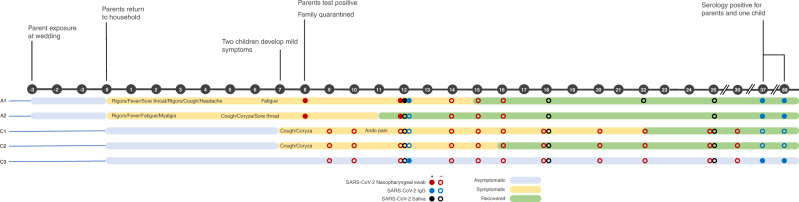
Timeline of travel, exposure, symptoms, and selected results. Nasopharyngeal PCR, saliva, and serum antibodies are shown for each parent and child. Key events in the timeline are highlighted according to anumber of days following the return of parents to the household.

Two parents (mother 38 years, and father 47 years) residing in Melbourne, Australia, attended a 3-hour wedding inter-state without their children, in early March 2020. They returned home 3-days later and developed cough, coryza, and subjective fevers, followed by lethargy and headache for a total of 14 (mother, A1) and 11 days (father, A2) (Fig. 1). Seven days after the onset of the parents’ symptoms, child one (male 9 years, C1) developed a mild cough, coryza, sore throat, abdominal pain, and loose stools, and child 2 (male 7 years, C2) developed mild cough and coryza. The third child (female 5 years, C3) was asymptomatic. Eight days after the onset of the parents’ symptoms, they were notified of an emerging outbreak of SARS-CoV-2 traced to the wedding. The parents were SARS-CoV-2 PCR positive on nasopharyngeal (NP) swabs taken the same day. Repeated NP swabs from the children were negative for SARS-CoV-2. Physical distancing precautions were not feasible in the household. Child 3 had particularly close contact, sleeping in the parents’ bed throughout the period both parents were unwell. All family members recovered fully without requiring medical care.

### SARS-CoV-2 testing

Serial samples, including blood, saliva, NP swabs, feces, and urine, were collected from all family members approximately every 2–3 days (Fig. 1). Daily symptoms were recorded in a standardized diary. Nasopharyngeal swabs from the parents on days 8 and 12 were SARS-CoV-2 PCR positive. All NP, saliva, and stool samples from the children were PCR negative for SARS-CoV-2. Nasopharyngeal swabs from the children were all positive for rhinovirus by a multiplex respiratory viral panel on day 10.

### Children and parents show an active cellular immune response

**Fig. 2:**
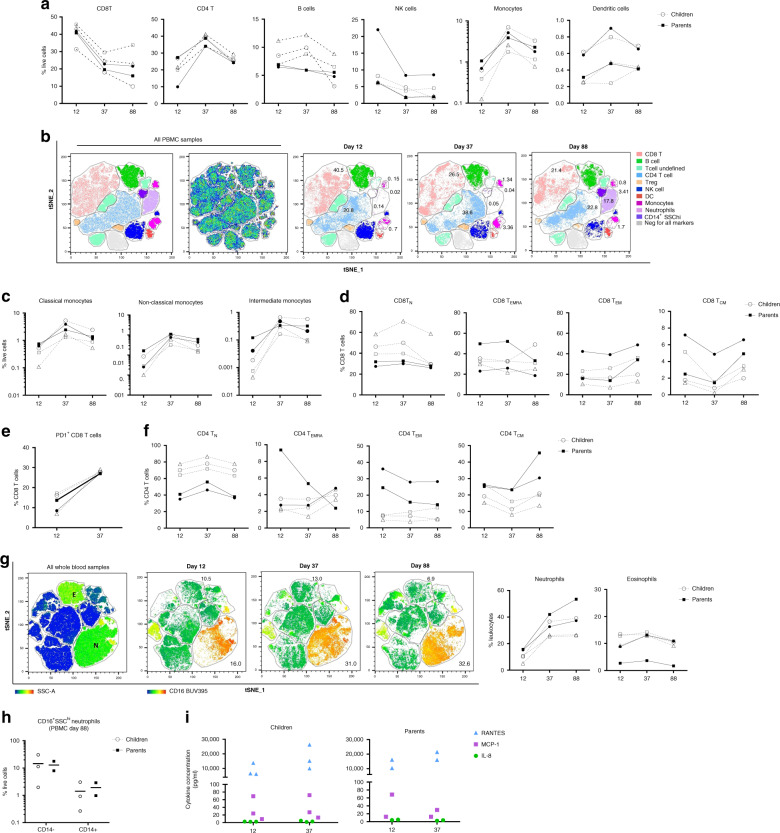
Longitudinal cellular immune profiling in parents and children. **a** Major immune cell populations in PBMC at day 12, 37, and 88 in parents (solid line) and children (broken line) (A1 (closed circles), A2 (closed squares), C1 (open circles), C2 (open squares), C3 (open triangles)). **b** tSNE dimensionality reduction of immune cell populations in all PBMC samples across the three-time points. The tSNE plot was generated from a concatenated file containing 300,000 events (20,000 randomly selected live single cells per patient per time point). **c**. Frequency of monocyte subpopulations in PBMC from parents and children. **d** Frequency of CD8 T cell naive, effector, and memory subpopulations in PBMC. **e** Frequency of PD1 expressing CD8 T cells over time. **f** Frequency of CD4 T cell naive, effector, and memory subpopulations in PBMC. **g** tSNE dimensionality reduction of whole blood samples. The tSNE plot was generated from a concatenated file containing 300,000 events (20,000 randomly selected live single cells per patient per time point). Coloring depicts SSC and CD16 expression in tSNE islands. Granulocyte populations (neutrophils and eosinophils) are expressed as the proportion of leukocytes. **h** Frequency of low-density CD16^+^SSC^hi^ neutrophils (CD14^+^ and CD14^−^) in PBMC fraction at day 88. **i** Plasma cytokine concentration of three detectable cytokines, RANTES (blue), MCP-1 (purple), and IL-8 (green) in children and parents at day 12 and 37.

We investigated the cellular immune response in peripheral blood mononuclear cells (PBMCs) from all family members on days 12, 37, and 88 by flow cytometry. Both parents and children had high proportions of CD8 T cells at day 12 that subsequently decreased (Fig. 2a), a decline associated with a corresponding increase in the proportion of CD4 T cells in all samples. Strikingly low proportions of monocytes were observed on day 12 in all family members, particularly in C3 (0.12%) relative to her siblings (average 0.5%) and parents (average 0.88%) (Fig. 2a). Monocytes returned to circulating proportions in all family members by day 37 (average 4.1%) and day 88 (average 2.5%). These signatures were also identified by unsupervised t-distributed stochastic neighbor embedding (tSNE) dimensionality reduction, where tSNE clusters corresponding to CD8 T, CD4 T, and monocytes in parents and children showed identical sequential changes to those observed by manual gating (Fig. 2b). Low proportions of monocytes were observed in all circulating subsets with reductions in CD16^+^ subsets most evident (Fig. 2c). Both parents showed increases in central (T_CM_) and effector (T_EM_) memory CD8 T cells by day 88 (Fig. 2d), and CD8 T cell expression of the exhaustion marker PD1 increased in all family members over time (Fig. 2e). CD4 T_EM_ cells reduced over time in the parents, and one parent (A2) had a marked decline in the CD4 effector (T_EMRA_) cell population (Fig. 2f). The heterogeneous cellular immune responses observed in all family members at the first timepoint are consistent with emerging evidence on SARS-CoV-2 infection in adults, including broad changes in the frequency, phenotype, and activation status of CD8 and CD4 T cells^14^. Depletion of innate immune cell subsets, including monocytes (particularly CD16^+^ subsets) has also been observed in COVID-19^15^.

A study comparing the blood and lung profiles of patients with severe COVID-19 revealed that non-classical monocytes preferentially migrate from the blood into the lungs during disease^16^. We observed further alterations in the myeloid compartment in our whole blood analysis. Low proportions of neutrophils were evident in all family members at day 12, particularly in C3 (5.1%) relative to her siblings (average 10.4%) and parents (average 15.5%) (Fig. 2g). Circulating neutrophils returned to an average of 30.5% in children and 45.4% in parents by day 88, a time point associated with the appearance of low-density immature neutrophils (SSC^hi^CD16^+^CD14^+/−^) in PBMCs of all family members (Fig. 2b, h). Pre- and immature-neutrophils in PBMC fractions have been recently described in SARS-CoV-2 infected adults^17^. In our study, parent A1 and all children had high proportions of eosinophils at all time points (Fig. 2g), in keeping with elevated eosinophils in SARS-CoV-2 infected patients during the recovery phase. Their role remains unclear^18^.

Our analyses highlighted that active cellular immune responses in the family members were not accompanied by a corresponding increase in plasma cytokine levels, consistent with mild or absence of symptoms. We quantified 18 plasma cytokines using a custom multiplex bead array and only IL-8, MCP-1, and CCL5 (RANTES) were detectable (Fig. 2i), with levels remaining constant over time, excluding C1 and C2 who had a ~2-fold increase in RANTES levels at day 37 (Fig. 2i). A case of mild adult COVID-19 disease reported an identical plasma cytokine signature to that observed in our family members^12^. RANTES has also recently been shown to be elevated in patients with both mild and severe COVID-19. In critically ill patients, blocking this pathway with a CCR5-targeted monoclonal antibody resulted in the restoration of T cell counts and reduced SARS-CoV-2 viral load^19^.

### SARS-CoV-2 antibodies in the saliva of all family members

**Fig. 3:**
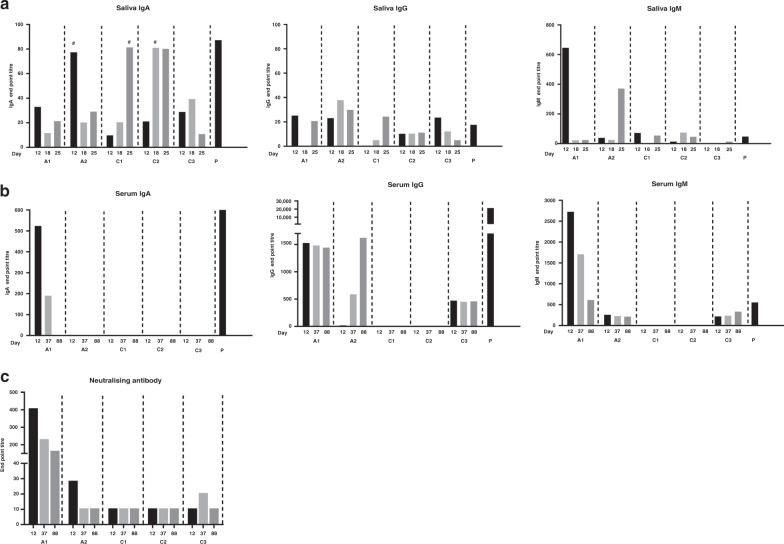
Salivary and plasma antibody responses against SARS-CoV-2 S1 protein by ELISA and by microneutralization assay. **a** Anti-S1 salivary IgA, IgG, and IgM. # IgA anti-S1 response that developed concurrent with resolution of symptoms. **b** Anti-S1 plasma IgA, IgG, and IgM. **c** Neutralizing antibody activity in plasma. A1: mother, A2: father, C1: male (9 years), C2: male (7 years), C3: female (5 years), (P) positive control.

To explore SARS-CoV-2 specific humoral immune responses, we first quantified salivary and plasma antibodies against the S1 protein by ELISA. Saliva from all family members tested positive for IgA antibodies against the S1 protein at all timepoints (Fig. 3a). A2 had high levels of salivary anti-S1 IgA at day 12, one day after symptom resolution. C1 and C2 also had increased anti-S1 salivary IgA (Fig. 3a; day 25 and day 18 samples, respectively), coincident with symptom resolution. Anti-S1 IgM and IgG were present in most salivary samples, but with a less consistent pattern in family members. Both parents and C3 had detectable levels of plasma IgG and IgM to SARS-CoV-2 S1 protein at all timepoints (Fig. 3b). IgG levels increased between timepoints for parent A2; those for parent A1 remained stable. Levels of S1-specific IgA in plasma were only detected in A1. Finally, A1 had a robust neutralizing antibody response on days 12, 37, and 88 (titers 403, 226, and 160, respectively) (Fig. 3c). A2 and C3 had a low level but detectable neutralizing antibody activity in sera on days 12 and 37, respectively. Serological responses to SARS-CoV-2 are associated with disease severity^20,21^, although data from children who often have milder disease or are likely to be asymptomatic are limited. In our study, children had low to undetectable serum antibodies while both parents had strong serum IgG responses out to day 88. More studies are needed to determine antibody magnitude and kinetics in children, including those with mild disease.

### Serological evidence of SARS-CoV-2 immunity

**Fig. 4:**
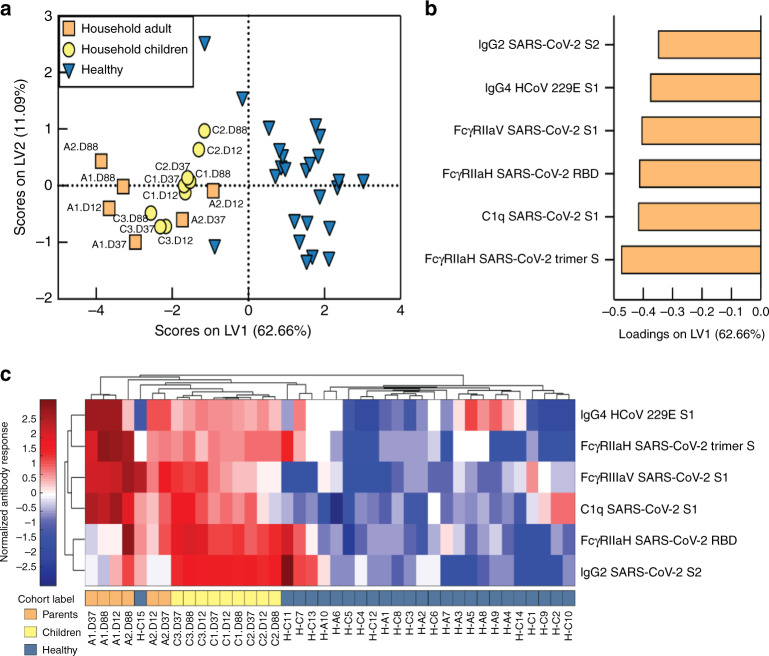
Family of symptomatic SARS-CoV-2 PCR positive parents and SARS-CoV-2 PCR negative children have distinct serological responses compared to healthy individuals, characterized by elevated SARS-CoV-2 specific responses. **a** PLSDA scores plot of healthy (blue triangles) vs family (circles) containing both SARS-CoV-2 PCR positive parents (orange) and negative children (yellow) exhibited 98.0% calibration and 96.0% cross-validation accuracy, with 62.7% of variance explained by LV1 (x-axis). Family member samples are labeled with A (adult) or C (child) with the day of sample collection listed after D. **b** PLSDA plot of LV1 loadings driving the separation of groups, where negatively loaded features are associated with the family members. **c** Hierarchical clustering of healthy individuals (blue) and family members (parents, orange; children, yellow) using a feature-selected serological signature, where red indicates a relatively high antibody response and blue a relatively low antibody response (z-score). Samples (x-axis) are labeled with H (healthy non-household members), and A (adult) or C (child). Day of sample collection is listed at the end of family member sample labels.

To further characterize whether the children had serological evidence of SARS-CoV-2 immunity despite being PCR negative, we undertook a systems serology analysis using a CoV-specific multiplex panel with the inclusion of additional aged-matched pre-pandemic healthy individuals. All family members, including the children, exhibited SARS-CoV-2-specific antibody features that differed from pre-pandemic controls (Fig. 4). This included serological signatures against the S1 protein, as well SARS-CoV-2 Trimer S, receptor-binding domain (RBD), and S2 with enhanced ability to bind Fcγ receptors or complement (C1q), suggesting that exposure to SARS-CoV-2 within this family induced Fc functionally active IgG subclass profiles. In addition, both parents, but not the children, had serological responses to other non-SARS-CoV-2 coronaviruses (Fig. 4c). Unsupervised hierarchical clustering analysis revealed that C3 clustered closest to her parents in all responses. C1 and C2, who had no evidence of a serologic response, clustered closest to the healthy controls whilst still exhibiting a SARS-COV-2 positive signature (Fig. 4c).

Our combined salivary and serological findings show that, despite having no virological evidence of infection, all three children developed antibody responses against various SARS-CoV-2 epitopes. Of the three children, C3, who remained asymptomatic throughout, demonstrated the most robust antibody response. We also observed that symptom resolution in A2, C1, and C2 coincided with a spike in salivary anti-S1 IgA, but not IgG. SARS-CoV-2 likely infects the salivary glands and is detectable in saliva^22^. Our data, therefore, provide evidence that control of SARS-CoV-2 at the site of infection may be mediated by a mucosal IgA antibody response. This potential key role for mucosal antibodies in protection warrants confirmation in larger studies. The timing of SARS-CoV-2 PCR testing occurred while 2 of 3 children were symptomatic and on multiple specimens, reducing the possibility that they cleared the infection prior to testing. Whilst rhinovirus was identified in the children’s respiratory panel, this is a common finding at our hospital and reflects recent exposure. The SARS-CoV-2 specific response identified in the saliva and serum would not be explained by this finding.

This in-depth family case study provides novel insights into immunological responses in children exposed to SARS-CoV-2. Despite close contact with infected parents, PCR testing for SARS-CoV-2 was repeatedly negative in all children, who developed minimal or no symptoms. However, the children had similar cellular and SARS-CoV-2 specific antibody-mediated immune responses to their parents, suggesting that the children were infected with SARS-CoV-2 but, unlike the adults, mounted an immune response that was highly effective in restricting virus replication. Whether this family will be protected from reinfection with SARS-CoV-2 is uncertain, as only one parent demonstrated a robust neutralizing antibody response. However, potent antiviral RBD-specific antibodies from memory B cell clones have been identified from convalescent patients with undetectable serum neutralizing responses^23^, raising the possibility that the children in our study may also have some degree of protection. The discordance between the virological PCR results and clinical serological testing, despite an evident immune response, highlights limitations to the sensitivity of nasopharyngeal PCR and current diagnostic serology in children. Our findings emphasize the need for further detailed investigation of the immune response to SARS-CoV-2 to advance our understanding of exposure and protective immunity in children.

## Methods

### SARS-CoV-2 detection

RNA was manually extracted from 140 μL of NP swabs and saliva, 280 μL of urine, and plasma and 140 μL of 20% (w/v) fecal suspension^24^ and then eluted in 50 to 60 μL sterile, molecular water (Life Technologies, Australia), using the QIAamp viral RNA kit (QIAgen GmbH, Hilden, Germany) according to the manufacturer’s instructions. A previously published RT-PCR protocol targeting the RdRp gene was used on an ABI 7500^25^. The nucleotide sequences of primers and probe are shown in Supplementary Table 2. SARS-CoV-2 standard (Exact Diagnostic, US) was used as a positive control for the PCR. Respiratory panel testing was by Ausdiagnostic viral panel.

### Plasma S1 and RBD ELISA

The ELISA method used to measure IgG, IgM, and IgA levels to SARS-COV-2 S1 and RBD protein was based on Amanat et al.^26^. Briefly, 96-well high-binding plates (Thermo Fisher Scientific) were coated with S1 or RBD (Sino Biological) diluted in PBS at 2 µg/mL and then incubated at 4 °C overnight. The following day, plates were washed with PBS containing 0.1% (v/v) Tween20 (PBS-T) and blocked with PBS containing 0.1% Tween and 10% (w/v) skim milk (PBS-TSM) for 1 h at room temperature (RT). Serial dilutions (3-fold) of plasma samples were prepared in PBS-TSM starting at 1:50. A positive control (convalescent sample) and negative control (pre-pandemic) sample were used in all assays. The blocking solution was removed and 100 µl of each serial dilution was added to the plates for 2 h at RT. The plates were then washed three times with 200 µl per well of PBS-T. Goat anti-human IgG- (1:10,000) or IgM- (1:5,000) horseradish peroxidase (HRP) conjugated secondary antibody (Southern Biotech) was prepared in PBS-TSM, and 50 µl of this secondary antibody was added to each well for 1 h. For IgA, 50 µL of biotinylated IgA (1:5000) was diluted in PBS-T and added to each well for 1 h, followed by the addition of Streptavidin-HRP to each well for 30 min. Plates were washed with PBS-T followed by distilled water and 50 µL of 3.3′, 5.5′-tetramethylbenzidine (TMB, Sera Care) substrate solution was added for 9 min. The reaction was stopped by the addition of 50 µL of 1 M phosphoric acid and optical densities measured using a microplate reader (Bio-Tek) at 450 nm (630 nm reference filter). Endpoint titers were calculated following background correction of the respective negative control reactivity in each assay.

### Saliva S1 protein ELISA

Saliva pooled under the tongue was drooled into a 50 mL tube and stored at −80 °C until analyzed. Immuno MaxiSorp 96-well ELISA plates (Thermo Fisher Scientific) were coated overnight at 4 °C with 2 µg/mL recombinant SARS-CoV-2/2019-nCoV S1 protein (Sino Biologicals) diluted in PBS. Wells were blocked with 10% skim milk in PBST (PBS + 0.1% Tween 20) at room temperature for 1 h. Two-fold serial dilutions of saliva samples in PBST were transferred to the ELISA plates (in duplicate) and incubated at room temperature for 1 h. Saliva from an asymptomatic individual confirmed negative for SARS-CoV-2 by clinical testing was used as a negative control. Saliva from a convalescent individual recently infected with SARS-CoV-2 was used as a positive control. Antibody binding was detected with biotinylated anti-human IgA (1:5000; Sigma-Aldrich) and IgG (1:10,000; Assay Matrix) for 1 hour at room temperature, then Streptavidin-HRP (1:5000; Life technologies) in PBST for 45 min at room temperature. Color was developed with TMB solution (Sigma-Aldrich) and H_2_O_2_ with the reaction stopped using 2 M H_2_SO_4_. Absorbance at 450 nm was read on a microplate reader. Examples of titrations are shown in Supplementary Fig. 2. OD values with negative control saliva were subtracted from the test samples at each dilution, then endpoint titers calculated.

### Microneutralisation assay

SARS-CoV-2 isolate CoV/Australia/VIC01/2020^27^ passaged in Vero cells was stored at −80 °C.

Serial two-fold dilutions of heat-inactivated plasma were incubated with 100 TCID_50_ of SARS-CoV-2 for 1 h and residual virus infectivity was assessed in quadruplicate wells of Vero cells; viral cytopathic effect was read on day 5. The neutralizing antibody titer is calculated using the Reed/Muench method^28,29^.

### Systems serology

*Healthy participants*. Age-matched children undergoing elective tonsillectomy (age 5–9) were recruited at the Launceston General Hospital (Tasmania) and, apart from fulfilling the criteria for tonsillectomy, they were considered otherwise healthy, showing no signs of immune compromise. Healthy adult donors (age 36–48) were recruited via the University of Melbourne. All healthy donors were recruited prior to SARS-CoV-2 pandemic. Heparinised blood was centrifuged for 10 min at 300 g to collect plasma, which was frozen at −20 °C until required.

*Coupling of carboxylated beads*. A custom CoV multiplex assay was designed^30^, with SARS-CoV-2 Spike 1 (Sino Biological), SARS-CoV-2 Spike 2, SARS-CoV Spike 1 (ACRO Biosystems, USA), and hCoV (229E, NL63, OC43) spikes (Sino Biologicals), as well as SARS-CoV-2 RBD (produced under HHSN272201400008C and obtained through BEI Resources, NIAID, NIH USA), SARS-CoV RBD (gift from Dale Godfrey) and both SARS-CoV-2 and HKU1 Trimeric Spikes (gift from Adam Wheatley). Tetanus toxoid (Sigma Aldrich) and influenza hemagglutinin (H1Cal2009; Sino Biological) were also added to the assay as positive controls. Antigens were covalently coupled to magnetic carboxylated beads (Bio Rad) using a two-step carbodiimide reaction and blocked with 0.1% BSA, before being resuspended and stored in PBS 0.05% sodium azide for use.

*Luminex bead-based multiplex assay*. The isotypes and subclasses of pathogen-specific antibodies present in collected plasma were assessed using the above multiplex assay^30^. Briefly, 20 µl of working bead mixture (1000 beads per bead region) and 20 µl of diluted plasma (final dilution 1:100) were added per well and incubated overnight at 4 °C on a shaker. Pathogen-specific antibodies were detected using 14 different detectors. One-step detection was done using phycoerythrin (PE)-conjugated mouse anti-human pan-IgG, IgG1-4, IgA1-2 (Southern Biotech; 1.3 µg/ml, 25 µl/well), where detectors were added to the beads, washed then read by the MagPix. C1q protein (MP Biomedicals, USA) was first biotinylated (Thermo Fisher Scientific, USA), then tetramerized with Streptavidin R-PE (SAPE; Thermo Fisher Scientific) before dimers or tetrameric C1q-PE were being used in one-step detection. For the detection of FcγR-binding, two-step detection was done by first adding soluble recombinant FcγR dimers (higher affinity polymorphisms FcγRIIa-H131, lower affinity polymorphisms FcγRIIa-R131, FcγRIIb, higher affinity polymorphisms FcγRIIIa-V158, lower affinity polymorphisms FcγRIIIa-F158; 1.3 µg/ml, 25 µl/well; gift from Bruce Wines and Mark Hogarth) to the beads, washing, followed by the addition of SAPE. Likewise, for IgM, two-step detection was done using biotinylated mouse anti-human IgM (mAb MT22; MabTech; 1.3 µg/ml, 25 µl/well;), followed by SAPE. Assays were repeated in duplicate.

*Data Pre-processing for Systems Serology Analysis*. In the multivariate analysis, positive control antigens (Tetanus and H1Cal2009) were removed. All visit days were used for each individual. Data was right-shifted and then log-transformed (log10(x + 1)). Right shifting was performed on each feature (detector-antigen pair) that contained negative values individually, by adding the minimum value for that feature to all samples within that feature. For all multivariate analysis the data were mean-centered and variance scaled for each feature using the z-score function in Matlab.

*Feature Selection*. To determine the minimal set of features (signatures) needed to classify the various cohorts, a three-step process was used based on^31^. First, the data were randomly sampled without replacement to generate 2000 subsets. All classes were resampled at the size of the smallest class for categorical outcomes, which corrected for any potential effects of class size imbalances during regularization. Elastic-Net regularization was then applied to each of the 2000 resampled subsets to select features most associated with cohort classifications. The Elastic-Net hyperparameter, alpha, was set to have equal weights between the L1 norm and L2 norm associated with the penalty function for the least absolute shrinkage and selection (LASSO) and ridge regression, respectively which allows for better analysis of collinear data, which may be eliminated in LASSO regression^32^. The frequency at which each feature was selected across the 2000 iterations was used to determine the signatures by using a sequential step-forward algorithm that iteratively added a single feature into a PLSDA model starting with the feature that had the highest frequency of selection, to the lowest frequency of selection. Model prediction performance was assessed at each step and evaluated by 10-fold cross-validation classification error. The model with the lowest classification error within a 0.01 difference between the minimum classification error was selected as the minimum signature. If only one feature was selected, the next best set of features was chosen. If consecutive feature sets were all equivalent, either the smallest or the largest set of features was chosen based on interpretability

*PLSDA*. Partial Least Squares Discriminant Analysis (PLSDA), performed in Eigenvectors PLS toolbox in Matlab, was used in conjunction with Elastic-Net, described above, to identify and visualize signatures that distinguish cohorts. This supervised method assigns a loading to each feature within a given signature, and identifies the linear combination of loadings (a latent variable) that best separates the categorical groups. A feature with a high loading magnitude indicates greater importance for separating the groups from one another. Each sample is then scored and plotted using their individual response measurements expressed through the latent variables (LVs). The scores and loadings can then be cross-referenced to determine which features are loaded in association with which categorical groups (positively loaded features are higher in positively scoring groups etc). All models are created with 10 fold cross-validation, where iteratively 10% of the data is left out as the test set, and the rest is used to train the model. Model performance is measured through calibration error (average error in the training set) as well as cross-validation error (average error in the test set), with values near zero being best. All models were othronogonalized to enable clear visualization of results.

*Hierarchical Clustering*. Cohort classification clustering was visualized for the Healthy vs. Household Cohort and based on their feature selected signatures described above, using unsupervised average linkage hierarchical clustering of z-scored data. Euclidean distance was used as the distance metric.

*Software*. PLSDA models were completed using the Eigenvector PLS toolbox in Matlab. Hierarchical Clustering was completed using MATLAB 2017b (MathWorks, Natick, MA). PLSDA scores and loadings plots were plotted in Prism version 8.0.0.

### Flow cytometry of PBMC and whole blood

Blood was collected in EDTA tubes from each participant at day 12, 37, and 88. Immediately following collection, 100 µl of whole blood was aliquoted for flow cytometry analysis. The remaining EDTA blood samples were processed into plasma and PBMC^33^. For flow cytometry analysis of whole blood samples, whole blood was lysed with 1 mL of red cell lysis buffer for 10 min at room temperature. Cells were washed with 1 mL PBS and centrifuged at 350 × *g* for 5 min. Following two more washes, cells were resuspended in PBS for viability staining using near infra-red viability dye according to manufacturer’s instructions. For flow cytometry analysis of freshly isolated PBMC, cells were washed in 1 mL PBS prior to viability staining using BV510 viability dye according to manufacturer’s instructions. For both whole blood and PBMC samples, the viability dye reaction was stopped by the addition of FACS buffer (2% heat-inactivated FCS in 2 mM PBS EDTA) and cells were centrifuged at 350 × *g* for 5 minutes. Cells were then resuspended in human FC-block according to manufacturer’s instructions for 5 min at room temperature. The whole blood or PBMC antibody cocktails (Supplementary Table 1) made up at 2× concentration were added 1:1 with the cells and incubated for 30 min on ice. Following staining, cells were washed with 2 mL FACS buffer and centrifuged at 350 × *g* for 5 min. Cells were then resuspended in 2% PFA for a 20 min fixation on ice, washed, and resuspended in 150 µl FACS buffer for acquisition using the BD LSR X-20 Fortessa. For all flow cytometry experiments, compensation was performed at the time of sample acquisition using compensation beads. Supplementary Fig. 1 depicts the manual gating strategy for PBMC and whole blood samples.

Results were analyzed (manual gating and tSNE analysis) using FlowJo Version 10.6 software. The tSNE plots were generated from a concatenated file containing 300,000 events (20,000 randomly selected live single cells per patient per time point). Manually gated results are presented as proportion of live cells or as proportion of parent gate (for PBMC) or as proportion of leukocyes (for whole blood). Data were plotted in Prism version 8.0.0.

### Plasma cytokines

Plasma was diluted 1:2 and 1:4 for assessment of cytokines using the human soluble protein cytometric bead array flex sets (BD Biosciences) according to manufacturer’s instructions. Cytometric bead array data were acquired on a BD LSR II X-20 Fortessa and analyzed using the FCAP Array Software. The following 18 cytokines were quantified: IL-1α, IL-1β, IL-6, IFNα, TNFα, MIP-1α, MCP-1, IL-8, RANTES, IL-12p70, IL-10, IL-2, IL-5, IL-5, IL-9, IL-13, IFNγ, and IL-17A. All cytokines except for IL-8, MCP-1, and RANTES fell below the limit of detection of the assay at both dilutions and were excluded from future analysis. Results are reported in pg/mL and plotted using Prism version 8.0.0.

### Ethics

Human experimental work was conducted according to the Declaration of Helsinki principles and according to the Australian National Health and Medical Research Council Code of Practice. All donors or their legal guardians provided written informed consent. The study was approved by the Human Research Ethics Committee (HREC) of the University of Melbourne (Ethics ID #1443389.4, #2056761, #1647326, #2056689, #1955465) for healthy adults, Tasmanian Health and Medical HREC (H0017479) for healthy child donors. For the family case study, this project received ethical approval from The Royal Children’s Hospital Melbourne Human Research Ethics Committee (HREC): HREC/63666/RCHM-2019.

### Reporting summary

Further information on research design is available in the Nature Research Reporting Summary linked to this article.

## Supplementary information

Supplementary Information

Reporting Summary

## Data Availability

All data supporting the findings of this study are available from the authors upon request. Source data are provided with this paper.

## Source data

Source Data
