# Supplementary material for: Immune responses to SARS-CoV-2 in three children of parents with symptomatic COVID-19
Source: Nat Commun. 2020 Nov 11;11:5703. doi: 10.1038/s41467-020-19545-8 (PMC7658256; doi:10.1038/s41467-020-19545-8)
Supplement: Supplementary file 1 — Supplementary Information [file 41467_2020_19545_MOESM1_ESM.pdf]

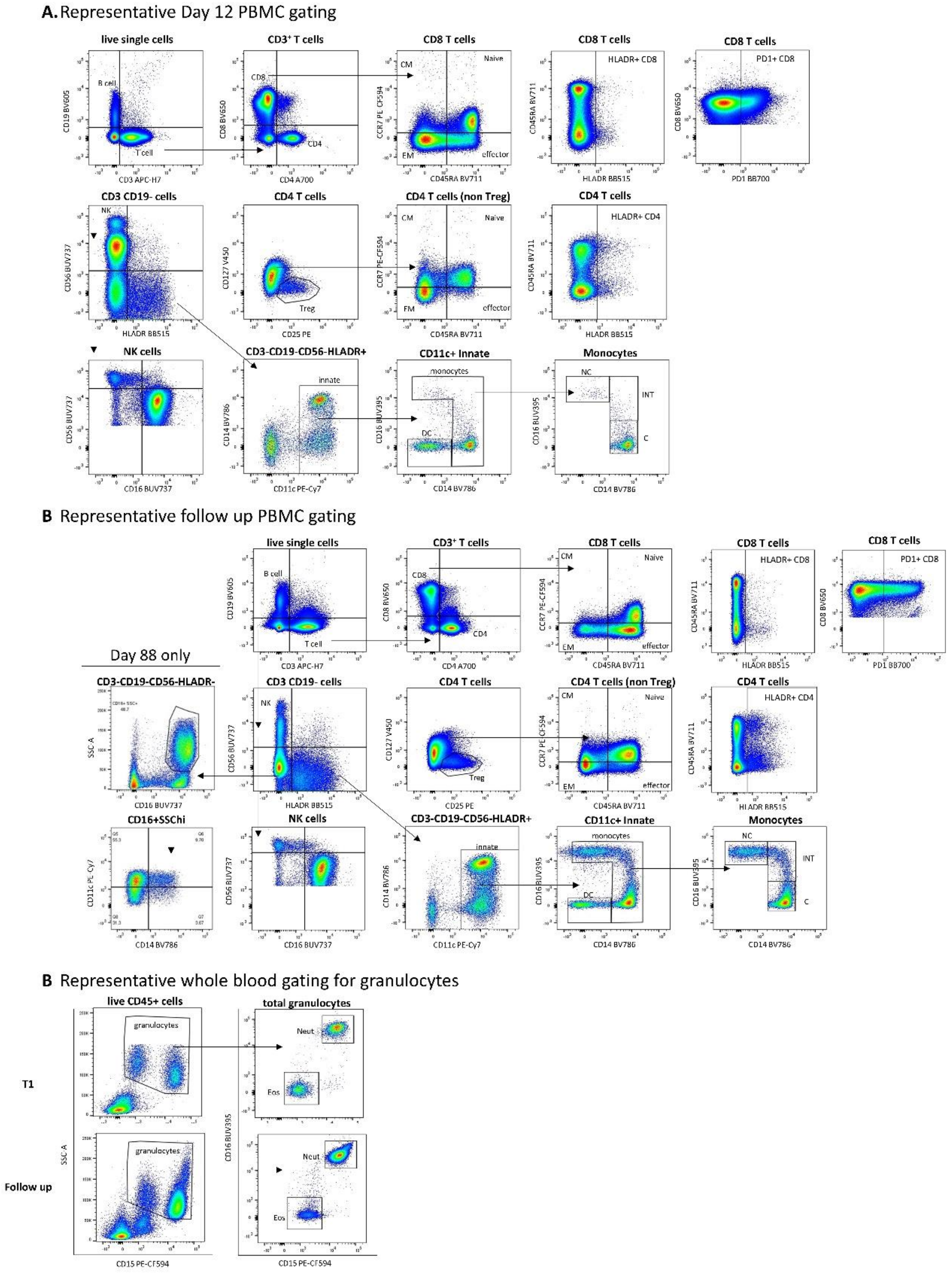

Supplementary Figure 1. Representative flow cytometry gating strategies for PBMC and whole blood at baseline and follow up. Within the PBMC fraction, B cells were selected based on CD19 expression, and the total T cell fraction based on CD3 expression. CD4 and CD8 T cells, and their naïve, effector, memory and regulatory (Treg) subsets were also quantified. HLADR^{+} and PD1^{+} T cells were investigated. CD3^{-}CD19^{-} cells were classified into NK cells (CD56^{+}) and innate cells (HLA-DR^{+}). Within the innate cell fraction, CD14^{+} monocytes and CD11c^{+} DCs were identified. Monocyte and NK cell subsets were identified based on CD16 expression. Low density neutrophils were observed in the PBMC fraction at day 88 only, characterised by a high SSC profile, CD16, CD14 and CD11c expression. For whole blood, granulocytes were selected within CD45^{+} leukocytes based on their SSC profile and CD15 expression. Neutrophils were CD15^{+}CD16^{+} and eosinophils were CD15^{+}CD16^{-}.

Supplementary Table 1. Flow cytometry antibody cocktails

**Surface Marker**	**Fluorophore**	**Clone**	**Final Dilution**
Whole blood panel			
CD14	BV786	M5E2	1:50
CD11b	BUV805	ICRF44	1:100
CD45	BV711	HI30	1:100
CD56	BUV737	NCAM16.2	1:100
CD11c	PE-Cy7	B-ly6	1:100
CD63	A647	H5C6	1:100
CD4	A700	RPA-T4	1:100
CD3	BB515	VCHTI	1:100
PD1	BB700	EH12.1	1:100
CD15	PE-CF594	W6D3	1:200
HLADR	V500	G46-6	1:200
CD19	BV605	5J25C1	1:200
CD8	BV650	RPA-T8	1:200
CD16	BUV395	3G8	1:400
PBMC panel			
CD25	PE	M-A251	1:25
CD127	V450	HIL7RM21	1:50
CD3	APCH7	SK7	1:50
CD14	BV786	M5E2	1:50
CD45RA	BV711	HI100	1:100
HLADR	BB515	G46-6	1:100
CD56	BUV737	NCAM16.2	1:100
CD11c	PE-Cy7	B-ly6	1:100
CD4	A700	RPA-T4	1:100
PD1	BB700	EH12.1	1:100
CCR7	PE-CF594	150503	1:200
CD19	BV605	5J25C1	1:200
CD8	BV650	RPA-T8	1:200
CD16	BUV395	3G8	1:400

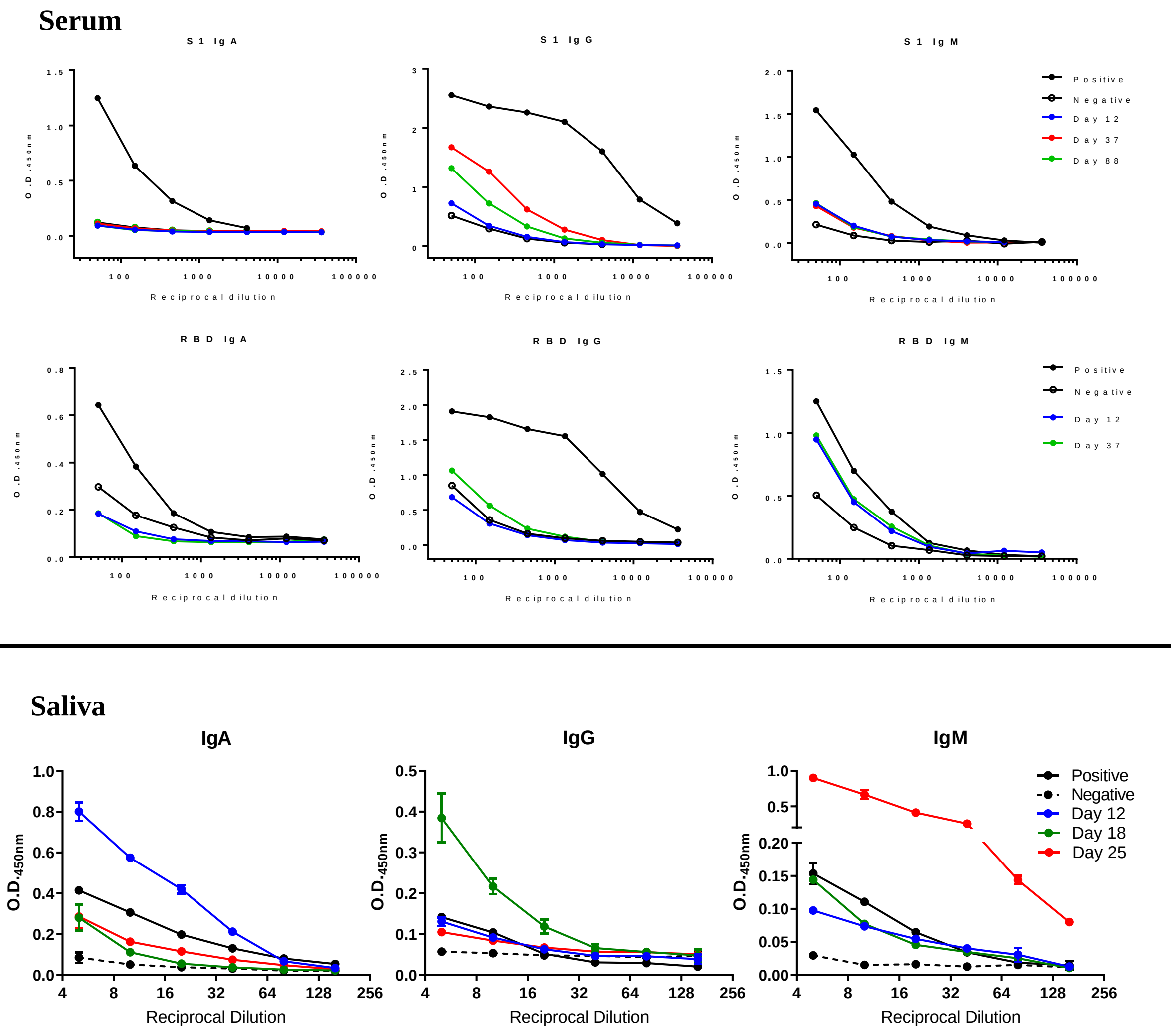

Supplementary Figure 2: Serum: Representative serum titration curves for the A2 family member on days 12, 37 and 88. A positive control sample from an adult convalescent patient and a pre-pandemic negative control is also shown. The top panel shows IgA, IgG and IgM responses to SARS-CoV-2 S1 domain of the Spike protein and the bottom panel show IgA, IgG and IgM responses to the receptor-binding domain (RBD). The day 88 sample was not available at the time of the RBD assay. **Saliva**: Representative titration curves of IgA, IgG and IgM ELISAs detecting antibodies against S1 protein in saliva. Data shown are the assay results for parent A2 from which end point titres were calculated. Where error bars are presented, they represent the mean ± standard deviation of two replicates. Broken line indicates the lack of anti-S1 antibody detection in negative control saliva.

Supplementary Table 2: Nucleotides sequences of primers and probe used for SARS-CoV-2 detection

NAME	SEQUENCE (5' to 3')
HK-RdRp/Hel-F	CGCATACAGTCTTRCAGGCT
HK-RdRp/Hel-R	GTGTGATGTTGAWATGACATGGTC
HK-RdRp/Hel-Probe	5' 6-FAM -TTAAGATGTGGTGCTTGCATACGTAGAC-3'BHQ1
