# Supplementary material for: Immune responses to SARS-CoV-2 in three children of parents with symptomatic COVID-19
Source: Nat Commun. 2020 Nov 11;11:5703. doi: 10.1038/s41467-020-19545-8 (PMC7658256; doi:10.1038/s41467-020-19545-8)
Supplement: Supplementary file 2 — Reporting Summary [file 41467_2020_19545_MOESM2_ESM.pdf]

nature research

Corresponding author(s): Shidan Tosif

Last updated by author(s): Jul 26, 2020

Reporting Summary

Nature Research wishes to improve the reproducibility of the work that we publish. This form provides structure for consistency and transparency in reporting. For further information on Nature Research policies, see our Editorial Policies and the Editorial Policy Checklist.

Statistics

For all statistical analyses, confirm that the following items are present in the figure legend, table legend, main text, or Methods section.

n/a	Confirmed	
☐	☒	The exact sample size (n) for each experimental group/condition, given as a discrete number and unit of measurement
☐	☒	A statement on whether measurements were taken from distinct samples or whether the same sample was measured repeatedly
☐	☒	The statistical test(s) used AND whether they are one- or two-sided<br>*Only common tests should be described solely by name; describe more complex techniques in the Methods section.*
☐	☒	A description of all covariates tested
☐	☒	A description of any assumptions or corrections, such as tests of normality and adjustment for multiple comparisons
☐	☒	A full description of the statistical parameters including central tendency (e.g. means) or other basic estimates (e.g. regression coefficient) AND variation (e.g. standard deviation) or associated estimates of uncertainty (e.g. confidence intervals)
☐	☒	For null hypothesis testing, the test statistic (e.g. F, t, r) with confidence intervals, effect sizes, degrees of freedom and P value noted<br>*Give P values as exact values whenever suitable.*
☒	☐	For Bayesian analysis, information on the choice of priors and Markov chain Monte Carlo settings
☐	☒	For hierarchical and complex designs, identification of the appropriate level for tests and full reporting of outcomes
☐	☒	Estimates of effect sizes (e.g. Cohen's d, Pearson's r), indicating how they were calculated

Our web collection on statistics for biologists contains articles on many of the points above.

Software and code

Policy information about availability of computer code

Data collection	REDCap V10.1.2, Microsoft Excel 2009 Office 365, BD FACS DIVA V9.0
Data analysis	Flowjo V10, tSNE plugin within Flowjo V10, Prism V8, PLSDA models were completed using the Eigenvector PLS toolbox in Matlab. Hierarchical Clustering was completed using MATLAB 2017b (MathWorks, Natick, MA). PLSDA scores and loadings plots were plotted in Prism version 8.0.0.

For manuscripts utilizing custom algorithms or software that are central to the research but not yet described in published literature, software must be made available to editors and reviewers. We strongly encourage code deposition in a community repository (e.g. GitHub). See the Nature Research guidelines for submitting code & software for further information.

Data

Policy information about availability of data

All manuscripts must include a data availability statement. This statement should provide the following information, where applicable:

- Accession codes, unique identifiers, or web links for publicly available datasets
- A list of figures that have associated raw data
- A description of any restrictions on data availability

The source data underlying all figures and supplemental data will be provided as a Source Data file

Field-specific reporting

Please select the one below that is the best fit for your research. If you are not sure, read the appropriate sections before making your selection.

☒ Life sciences ☐ Behavioural & social sciences ☐ Ecological, evolutionary & environmental sciences

For a reference copy of the document with all sections, see nature.com/documents/nr-reporting-summary-flat.pdf

Life sciences study design

All studies must disclose on these points even when the disclosure is negative.

Sample size	The case study consists of five family members, each with samples collected at three time points. 25 aged matched healthy controls were used to generate the data in figure 4. The family of five was chosen as a case report due to discordance of COVID-19 between adults and children.
Data exclusions	No data were excluded from the analysis
Replication	Where biological sample volume permitted, each test was replicated at least once to verify all findings. All attempts at replication were successful.
Randomization	This was a case study where the family was selected based on their COVID-19 clinical outcomes and as such there was no randomization for participants
Blinding	Investigators were blinded to disease outcome during experimentation and data analysis.

Reporting for specific materials, systems and methods

We require information from authors about some types of materials, experimental systems and methods used in many studies. Here, indicate whether each material, system or method listed is relevant to your study. If you are not sure if a list item applies to your research, read the appropriate section before selecting a response.

Materials & experimental systems

n/a	Involved in the study	
☐	☒	Antibodies
☒	☐	Eukaryotic cell lines
☒	☐	Palaeontology and archaeology
☒	☐	Animals and other organisms
☐	☒	Human research participants
☒	☐	Clinical data
☒	☐	Dual use research of concern

Methods

n/a	Involved in the study	
☒	☐	ChIP-seq
☐	☒	Flow cytometry
☒	☐	MRI-based neuroimaging

Antibodies

Antibodies used

Surface Marker Fluorophore Clone Final Dilution
Whole blood panel (all from BD Biosciences)
CD14 BV786 M5E2 1:50
CD11b BUV805 ICRF44 1:100
CD45 BV711 HI30 1:100
CD56 BUV737 NCAM16.2 1:100
CD11c PE-Cy7 B-ly6 1:100
CD63 A647 H5C6 1:100
CD4 A700 RPA-T4 1:100
CD3 BB515 VCHTI 1:100
PD1 BB700 EH12.1 1:100
CD15 PE-CF594 W6D3 1:200
HLADR V500 G46-6 1:200
CD19 BV605 5J25C1 1:200
CD8 BV650 RPA-T8 1:200
CD16 BUV395 3G8 1:400
PBMC panel (all from BD Biosciences)
CD25 PE M-A251 1:25
CD127 V450 HIL7RM21 1:50

	CD3 APCH7 SK7 1:50<br>CD14 BV786 M5E2 1:50<br>CD45RA BV711 HI100 1:100<br>HLADR BB515 G46-6 1:100<br>CD56 BUV737 NCAM16.2 1:100<br>CD11c PE-Cy7 B-ly6 1:100<br>CD4 A700 RPA-T4 1:100<br>PD1 BB700 EH12.1 1:100<br>CCR7 PE-CF594 150503 1:200<br>CD19 BV605 5J25C1 1:200<br>CD8 BV650 RPA-T8 1:200<br>CD16 BUV395 3G8 1:400
Validation	Each lot of conjugated antibody is quality control tested by flow cytometry analysis of stained cells using the appropriate positive and negative cell staining and/or activation controls (see supplemental figure 1). All antibodies are mouse anti-human, and were tested on human cells.

Human research participants

Policy information about studies involving human research participants

Population characteristics	One family of five were involved in this study. Two parents (A1 - mother 38 years, and A2 - father 47 years), child one (male 9 years, C1) child 2 (male 7 years, C2) and child 3 (female 5 years, C3).
Recruitment	Patients were recruited through the Respiratory Infection Clinic at the Royal Children's Hospital in March 2020. Criteria for recruitment was a positive COVID-19 case in the household.
Ethics oversight	This project received ethical approval from The Royal Children's Hospital Melbourne Human Research Ethics Committee (HREC): HREC/63666/RCHM-2019.

Note that full information on the approval of the study protocol must also be provided in the manuscript.

Flow Cytometry

Plots

Confirm that:

- [x] The axis labels state the marker and fluorochrome used (e.g. CD4-FITC).
- [x] The axis scales are clearly visible. Include numbers along axes only for bottom left plot of group (a 'group' is an analysis of identical markers).
- [x] All plots are contour plots with outliers or pseudocolor plots.
- [x] A numerical value for number of cells or percentage (with statistics) is provided.

Methodology

Sample preparation	Blood was collected in EDTA tubes from each participant at day 12, 37 and 88. Immediately following collection, 100µl of whole blood was aliquoted for flow cytometry analysis. The remaining EDTA blood samples were processed into plasma and PBMC as previously described 28. For flow cytometry analysis of whole blood samples, whole blood was lysed with 1mL of red cell lysis buffer for 10 minutes at room temperature. Cells were washed with 1mL PBS and centrifuged at 350 x g for 5 minutes. Following two more washes, cells were resuspended in PBS for viability staining using near infra-red viability dye according to manufacturers instructions. For flow cytometry analysis of freshly isolated PBMC, cells were washed in 1mL PBS prior to viability staining using BV510 viability dye according to manufacturers instructions. For both whole blood and PBMC samples, the viability dye reaction was stopped by the addition of FACS buffer (2% heat-inactivated FCS in 2mM EDTA) and cells were centrifuged at 350 x g for 5 minutes. Cells were then resuspended in human FC-block according to manufacturers instructions for 5 minutes at room temperature. The whole blood or PBMC antibody cocktails (Extended data Table 1) made up at 2X concentration were added 1:1 with the cells and incubated for 30 minutes on ice. Following staining, cells were washed with 2mL FACS buffer and centrifuged at 350 x g for 5 minutes. Cells were then resuspended in 2% PFA for a 20 minute fixation on ice, washed, and resuspended in 150µl FACS buffer for acquisition using the BD LSR X-20 Fortessa. For all flow cytometry experiments, compensation was performed at the time of sample acquisition using compensation beads.
Instrument	BD Fortessa X-20 (special order research product)
Software	Data collection: DIVA<br>Data analysis: FlowJo V10
Cell population abundance	No cell populations were sorted.
Gating strategy	Within the PBMC fraction, B cells were selected based on CD19 expression, and the total T cell fraction based on CD3 expression. CD4 and CD8 T cells, and their naïve, effector, memory and regulatory (Treg) subsets were also quantified. HLADR

+ and PD1+ T cells were investigated. CD3-CD19- cells were classified into NK cells (CD56+) and innate cells (HLA-DR+). Within the innate cell fraction, CD14+ monocytes and CD11c+ DCs were identified. Monocyte and NK cell subsets were identified based on CD16 expression. Low density neutrophils were observed in the PBMC fraction at day 88 only, characterised by a high SSC profile, CD16, CD14 and CD11c expression. For whole blood, granulocytes were selected within CD45+ leukocytes based on their SSC profile and CD15 expression. Neutrophils were CD15+CD16+ and eosinophils were CD15+CD16-. This is shown in detail in Extended Data Figure 1.

☒ Tick this box to confirm that a figure exemplifying the gating strategy is provided in the Supplementary Information.
